# Blockade LAT1 Mediates Methionine Metabolism to Overcome Oxaliplatin Resistance under Hypoxia in Renal Cell Carcinoma

**DOI:** 10.3390/cancers14102551

**Published:** 2022-05-22

**Authors:** Qingwen Xu, Yuxi Liu, Wen Sun, Tiantian Song, Xintong Jiang, Kui Zeng, Su Zeng, Lu Chen, Lushan Yu

**Affiliations:** 1 Zhejiang Province Key Laboratory of Anti-Cancer Drug Research, Institute of Drug Metabolism and Pharmaceutical Analysis, College of Pharmaceutical Sciences, Zhejiang University, Hangzhou 310058, China; qingwenxu@zju.edu.cn (Q.X.); liuyuxi@zju.edu.cn (Y.L.); sunw127@zju.edu.cn (W.S.); 22119089@zju.edu.cn (T.S.); 22119049@zju.edu.cn (X.J.); huhaihong@zju.edu.cn (K.Z.); zengsu@zju.edu.cn (S.Z.); 2 Key Laboratory of Clinical Cancer Pharmacology and Toxicology Research of Zhejiang Province, Department of Clinical Pharmacology, Affiliated Hangzhou First Peoples Hospital, Cancer Center, Zhejiang University School of Medicine, Hangzhou 310006, China; 3 Westlake Laboratory of Life Sciences and Biomedicine of Zhejiang Province, Hangzhou 310024, China; 4 Department of Pharmacy, Second Affiliated Hospital, School of Medicine, Zhejiang University, Hangzhou 310009, China

**Keywords:** renal cell carcinoma, hypoxia, methionine metabolism, LAT1, drug combination

## Abstract

**Simple Summary:**

The transformation and mechanism of methionine metabolism of renal cell carcinoma (RCC) under a hypoxic microenvironment is not well understood as yet. This study illustrated that the reprogramming of methionine metabolism and the subsequent glutathione (GSH) synthesis were mediated by amino acid transporter 1 (LAT1). Correspondingly, we proposed a combination strategy of LAT1 inhibitor JPH203 and oxaliplatin, which presented an enhanced therapeutic efficacy for RCC both in vivo and in vitro.

**Abstract:**

Hypoxic microenvironment and metabolic dysregulation of tumor impairs the therapeutic efficacy of chemotherapeutic drugs, resulting in drug resistance and tumor metastasis, which has always been a challenge for the treatment of solid tumors, including renal cell carcinoma (RCC). Herein, starting from the evaluation of methionine metabolism in RCC cells, we demonstrated that the increased methionine accumulation in RCC cells was mediated by L-type amino acid transporter 1 (LAT1) under hypoxia. Glutathione (GSH), as a methionine metabolite, would attenuate the therapeutic efficacy of oxaliplatin through chemical chelation. Reducing methionine uptake by LAT1 inhibitor JPH203 significantly enhanced the sensitivity of RCC cells to oxaliplatin by reducing GSH production in vitro and in vivo. Therefore, we proposed an effective and stable therapeutic strategy based on the combination of oxaliplatin and LAT1 inhibitor, which is expected to solve the resistance of RCC to platinum-based drugs under hypoxia to a certain extent, providing a meaningful insight into the development of new therapeutic strategies and RCC treatment

## 1. Introduction

Renal cell carcinoma (RCC) is a heterogeneous group of urological malignancy which arises from renal tubular epithelial cells, accounting for 2–3% of all cancers in adults [1]. The mortality rate of RCC is about 2.5% of all cancers and the five-year survival rate of metastatic RCC is less than 8% [2,3]. In clinical practices, RCC patients still experience recrudescence or acquired resistance after undergoing targeted or immunological therapies [4,5,6]. Intensive studies have disclosed that tumor microenvironment (TME) plays a key role in drug resistance for various cancers [7,8]. A hypoxic microenvironment is typical characteristic of up to 50–60% of solid tumors including RCC [9], which is caused by abnormal vasculature and increased oxygen consumption by cancer cells [10]. Tumor cells in hypoxic TME normally presented a resistance profile to radiation and drug therapy because hypoxia mediates epigenetic reprogramming [11], hypoxia-inducible factors (HIF) production [12], drug efflux [13], and metabolic reprogramming [12]. In addition, since the metabolism of RCC is altered significantly (so-called metabolic reprogramming) for optimizing tumor energetics and biosynthesis, RCC has been labeled as a metabolic disease, which consequently provides opportunities for new therapeutic strategies, biomarkers, and imaging modalities [14,15,16]. Therefore, elucidating the hypoxic effect on metabolic reprogramming of RCC and exploring a stable strategy is of great value for promoting RCC treatment.

Methionine as a sulfur-containing essential amino acid involved epigenetic regulation, cell proliferation, signal transduction, nuclear functions, and redox homeostasis by mediating the molecular biology transition of RCC [17,18]. During methionine metabolism, methionine converts to S-adenosylmethionine (SAM) through adenylation, thereby promoting methylation modification and mTORC1 activity [19,20]. In addition, methionine also participates in synthesis of polyamines and glutathione (GSH), maintaining nuclear functions and redox homeostasis [21]. Due to the conspicuous roles in the mentioned mechanism, methionine is confirmed to be closely related to tumorigenesis, immune escape, and drug resistance [22,23]. Furthermore, dietary methionine restriction has been proposed as a strategy for various tumor treatment because of severe methionine dependence [24,25]. However, systemic restriction of the methionine by diet leads to negative effects on normal physiological activities, including dysfunction of gut flora and the immune system [24,26]. Therefore, focus on the upstream passage of methionine metabolism, investigating a novel tactics to limit the methionine level precisely in the RCC cells, may have a role in promoting the in-depth research of RCC and the development of new treatment methods.

Herein, starting from methionine metabolism in RCC, we found that amino acid transporter 1 (LAT1) plays a significant role in the transportation of methionine in hypoxic environments. Further work showed that GSH production could be promoted by accumulated methionine, which could be inhibited precisely by the LAT1 inhibitor JPH203. To elucidate the correlation between methionine metabolism and chemotherapeutic resistance in the hypoxic tumor microenvironment, this work proposed a novel therapeutic strategy based on combination of LAT1 inhibitor and oxaliplatin, which is expected to solve the resistance of RCC cells to platinum drugs, consequently providing an important insight into the treatment of RCC.

## 2. Materials and Methods

### 2.1. Cell Culture and Drug Treatment

Human renal cell adenocarcinoma cell lines (786-O and 769-P) were purchased from the Chinese Academy of Science Committee on type culture collection cell libraries (Shanghai, China). The 786-O and 769-P cell lines were cultured in RPMI 1640 medium (C11875500BT, Gibco, Gaithersburg, MD, USA) supplemented with 10% fetal bovine serum (FBS, 10099-141, Gibco, Gaithersburg, MD, USA), 1% penicillin/streptomycin (P1400, Solarbio, Beijing, China), and 1% sodium pyruvate (S8636, Sigma-Aldrich, St. Louis, MO, USA). Cells were maintained in a 5% CO_2_ cell incubator or in anaerobic incubator (Electrotek AW400SG, West Yorkshire, UK) to establish hypoxic environment (1% oxygen). For drug treatment, oxaliplatin (61825-94-3, Aladdin, Shanghai, China) and JPH203 (1597402-27-1, Aikon Biopharmaceutical, Nanjing, China) were dissolved in distilled water and DMSO, respectively. Then, the drug was added to the culture medium at the indicated doses.

### 2.2. Clinical Samples Collection

Matched human surgical specimens from renal cell carcinoma patients were obtained from Specimen Bank of Zhejiang Cancer Hospital (Hangzhou, China) with informed consent. All the sample collections were approved by Institutional Review Board of Zhejiang Cancer Hospital and comply with relevant regulations and ethical requirements (Ethics Code: IRB-2017-02). Tissue specimen information were listed in Supplementary Table S2.

### 2.3. Real-Time Quantitative PCR and Western Blotting

For real-time quantitative PCR (RT-qPCR), total RNA was isolated from cells using the AxyPrep^TM^ RNA Miniprep kit (AP-MN-MS-RNA-250, Axygen, Union City, CA, USA). Sample with 500 ng RNA was reversely transcribed into cDNA using PrimeScript^TM^ RT Master Mix kit (RR036A, Takara, Shiga, Japan). RT-qPCR was performed with TB Green Premix EX Taq^TM^ (RR420A, Takara, Shiga, Japan) in real-time PCR system (StepOnePlus, Applied Biosystems, Waltham, MA, USA). *GAPDH* was used as the normalizing gene. Specific primers are listed in Supplementary Materials Table S1.

For Western blot analysis, protein from tissues and cells was extracted and lysed with RIPA lysis buffer containing protease inhibitor PMSF (ST506, Beyotime, Shanghai, China), Leupeptin (A600580-0005, Beyotime, Shanghai, China), and Pepstatin (A610583-0005, Beyotime, Shanghai, China). Protein concentration was quantified using BCA protein assay kit (P0011, Beyotime, Shanghai, China). The protein was separated using 10% SDS-PAGE and transferred to polyvinylidene difluoride membrane (IPVH00010, Millipore, Burlington, MA, USA). The membrane was hybridized with HRP-conjugated secondary antibodies and visualized in G-BOX gel imaging system (Chemi XR 5, Syngene, Cambridge, UK) after being blocked with 5% fat-free milk and reacted with primary antibodies overnight at 4 °C. The information of antibodies and dilution as follows: anti-LAT1 (5347S, Cell Signaling Technology, Danvers, MA, USA) at 1:1000 dilution; anti-GAPDH (60004-1-Ig, Proteintech, Rosemont, IL, USA) at 1:5000 dilution; HRP-conjugated goat anti-mouse IgG (H + L) (70-GAM0072, Multi Sciences, Hangzhou, China) at 1:5000 dilution; HRP-conjugated goat anti-Rabbit IgG (H + L) (70-GAR0072, Multi Sciences, Hangzhou, China) at 1:5000 dilution.

### 2.4. Metabolomics Analysis

The 769-P cells were planted onto 15 cm dishes and cultured in normoxic (20% oxygen) and hypoxic (1% oxygen) conditions for 72 h, respectively. The cells were collected and washed by PBS buffer for three times, and then resuspended in cold PBS buffer. The metabolomics analysis was carried out by a service provider (Lianchuan, Hangzhou, China).

The accumulation of methionine was detected using liquid chromatography–mass spectrometry (LC-MS). The RCC cell lines (786-O and 769-P) were collected and washed by PBS buffer three times. Then, the samples were ultrasonicated for 3 min at 4 °C. After centrifugation (12,000 rpm, 10 min, 4 °C), 60 µL supernatant was transferred and 2.5 ng/mL voriconazole (V129745, Aladdin, Shanghai, China) was used as internal standard. The detailed parameters are described in the following.

Chromatographic conditions: Agilent ZORBAX Eclipse XDB-C18 (2.1 mm × 50 mm, particle size 3.5 µm). Phase A is ammonium acetate (10 mM) containing 0.1% formic acid and phase B is methanol containing 0.1% formic acid. The flow rate was 0.300 mL/min, the injection volume was 5.0 µL. Gradient elution as follows: 0–2 min, 95% A; 2–3 min, 95–5% A; 3–5 min, 5% A; 5–6 min, 95% A.

Mass spectrometry conditions: ESI^+^; MRM monitoring mode; ion source temperature: 150 °C; desolvation temperature: 450 °C; capillary voltage: 3 kV; cone voltage: 20 V; gasification gas flow: 5 L/min.

Quantitative ion pair: methionine *m/z* 149.97 → 73.92; voriconazolec *m/z* 350.16 → 126.89.

### 2.5. Construction of Knockdown and Overexpression RCC Cells

LAT1 knockdown and overexpression cells were constructed using the small interfering RNA (siRNA) and LAT1-CDS-3‘-UTR pCDH lentiviral plasmid, respectively. siRNA and lentiviral vector were purchased form Genomeditech (Shanghai, China). Under the guidance of protocol, the siRNAs or plasmid was transfected into RCC cells using Jet PRIME^®^ (114-01, Polyplus, Shanghai, China). The sequences (5′ to 3′) of siRNAs were as follows: siNC: UUCUCCGAACGUGUCACGUdTdT; siLAT1-1: GCAUUAUACAGCGGCCUCUUUdTdT; siLAT1-2: GCCGUGGACUUCGGGAACUAUdTdT.

### 2.6. Reduced Glutathione Assays

The RCC cell lines (769-P and 786-O) were collected and washed by PBS buffer for three times. After centrifugation at 600× *g* for 10 min, reduced glutathione was extracted using micro-reduced glutathione assay kit (BC1175, Solarbio, Beijing, China) and measured the absorbance (412 nm) using multifunctional microplate reader.

### 2.7. Platinum Quantification

The RCC cell lines (786-O and 769-P) were treated with 50 µmol/L oxaliplatin for 48 h after being co-incubated with JPH203 (0 and 10 µmol/L) for 48 h. Cells were washed with cold PBS buffer for three times, then we added 150 µL 0.1% sodium dodecyl sulfonate, with shaking for 15 min. Subsequently, 150 µL supernatant was transferred after centrifugation (13,000 rpm for 15 min). The samples were digested with 200 µL 68% (*w*/*w*) HNO_3_ and 400 µL 30% (*w*/*w*) H_2_O_2_ for 1.5 h at 85 °C. After cooling, 200 µL ammonia water was added to neutralize the excessive HNO_3_. Distilled water was added to make final volume 4 mL and filtered through 0.22 µm membrane filter. The amount of platinum in samples was determined by inductively coupled plasma mass spectrometry (ICP-MS).

### 2.8. Cell Viability Assay

The RCC cell lines (769-P and 786-O) were seeded in triplicates onto 96-well plates and maintained in cell incubator overnight. For the drug combination in vitro, the medium was replaced by fresh medium containing JPH203 at the gradient doses for 48 h followed by adding oxaliplatin with indicated doses for 48 h. In JPH203 or oxaliplatin alone groups, another drug was replaced by DMSO or distilled water. The cells were cultured in fresh medium with 10% CCK-8 (c6030, NCM Biotech, Suzhou, China) and incubated at 37 °C for 1.5 h. The absorbance was measured at 450 nm using a multifunctional microplate reader.

### 2.9. Animal Studies

Six-week-old female BALB/c nude mice with an average weight of 20 g were purchased from Beijing Vital River Laboratory Animal Technology Co., Ltd. (Beijing, China). During the experiment, the animals were housed in the SPF system of laboratory animal center of Zhejiang University (temperature: 19–29 °C, humidity: 40–70%, light time: 12 h, ventilation times number: 12–15 times/h, pressure gradient: 20–50 Pa, air velocity: 0.13–0.18 m/s).

We made all efforts to minimize animal suffering. All husbandry procedures and protocols were in accordance with “Guidelines for the Care and Use of Laboratory Animals” and were approved by Zhejiang University Animal Care and Use Committee. Ethics Code: ZJU20210163. 

The mice were injected subcutaneously with 1 × 10^7^ 786-O cells. The tumor volumes were measured with two dimensions (L: longest dimension; W: shortest dimension) and calculated as follows: L × W^2^/2. When the tumor volume reached 100 mm^3^, the mice were randomized to four group (*n* = 9). The animals were treated with three cycles of the following dosage regimen (either single drug or in combination): JPH203 was given intraperitoneally at a dose of 25 mg/kg for three days, then oxaliplatin was given intraperitoneally at a dose of 10 mg/kg in absence of JPH203. At day 14 of drug administration, three mice in each group were sacrificed and the tumors were stripped for detecting the amount of glutathione. The remnant mice were sacrificed at day 29. Then, the tissues of kidney and liver, tumor, and blood were collected to further analyzed. The weights and tumor volumes were monitored during the experiment.

### 2.10. Statistical Analyses

Statistical analysis was performed using Graphpad Prism 8 software (Graphpad Soft-wear, La Jolla, CA, USA). Results are representative of at least three independent experiments and all statistical analyses were expressed as mean ± SEM (standard error of the mean). Students *t*-test (two-tailed), one-way ANOVA analysis, and two-way ANOVA analysis were used in this study. A value of *p* < 0.05 was considered statistically significant. 

## 3. Results

### 3.1. Methionine Metabolism Is Induced in RCC Cells under Hypoxic Condition

**Figure 1:**
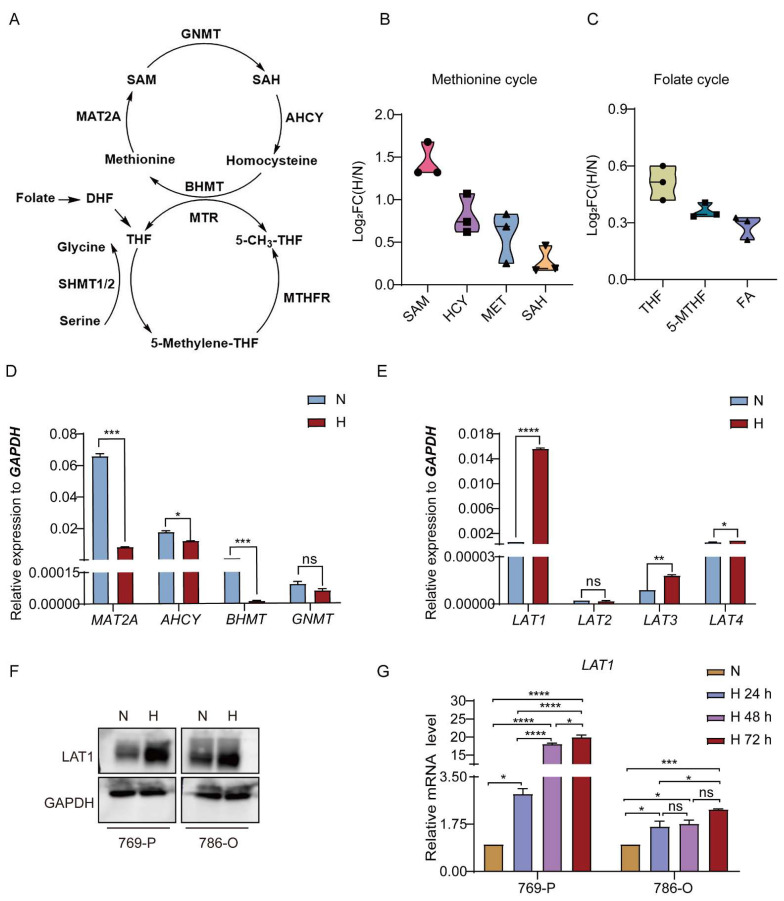
Increased methionine metabolism is mediated by LAT1 upregulation in RCC cells under hypoxia. (**A**) The schema of pivotal metabolites and enzymes in methionine cycle and folate cycle. (**B**) Relative amount of methionine (MET), homocysteine (HCY), S-adenosylhomocysteine (SAH), and S-adenosylmethionine (SAM) in 769-P cells cultured under normoxia and hypoxia for 72 h, respectively. (**C**) Relative abundance of tetrahydrofolate (THF), 5-methyl-tetrahydrofolate (5-MeTHF), and folate (FA) in 769-P cells cultured under normoxia and hypoxia for 72 h, respectively. (**D**) The mRNA expression of pivotal enzymes (MAT2A, AHCY, BHMT, and GNMT) of methionine cycle in 769-P cells cultured under normoxia and hypoxia for 72 h, respectively. MAT2A—Methionine adenosyltransferase 2A; GNMT—Glycine N-methyltransferase; AHCY—Adenosylhomocysteinase; BHMT—Betaine-homocysteine S-methyltransferase. (**E**) mRNA level of methionine uptake transporters (LAT1, LAT2, LAT3, and LAT4) in 769-P cells cultured under normoxia and hypoxia for 72 h, respectively. LAT—L-type amino acid transporter. (**F**) LAT1 protein expression in 769-P and 786-O cells cultured under normoxia and hypoxia for 72 h, respectively. (**G**) RT-qPCR was used to detect the mRNA expression of LAT1 in 786-O and 769-P cells exposing under hypoxic conditions for 0, 24, 48, and 72 h, respectively. FC—fold change; N—normoxia; H—hypoxia. GAPDH was used as an internal control. Data are the mean ± SEM for biological triplicates. Students *t*-test (two-tailed) and one-way ANOVA analysis were used. * *p* < 0.05; ** *p* < 0.01; *** *p* < 0.001; **** *p* < 0.0001. ns—no significance.

In order to explore the change of methionine metabolism in RCC cells under hypoxia, we detected the content of the intermediates of the methionine cycle and upstream folate cycle in 769-P cells cultured under normoxic condition (20% oxygen) and hypoxic conditions (1% oxygen), respectively (Figure 1A). As shown in Figure 1B, the abundances of methionine, homocysteine (HCY), S-adenosylhomocysteine (SAH), and S-adenosylmethionine (SAM) were all significantly increased (log_2_FC(H/N) > 0) in hypoxic conditions. In addition, the enrichment of intermediates of folate cycle was observed under hypoxia, which provided methyl units for homocysteine to complete the methionine cycle (Figure 1C).

To explore the reason that methionine metabolism was markedly vibrant under hypoxia, the pivotal enzymes involved in methionine cycle and folate cycle were first evaluated in 786-O and 769-P cells under normoxia and hypoxia, respectively (Figure 1D, Figures S1B and S2A). Glyceraldehyde-3-phosphate dehydrogenase (*GAPDH*) was selected as the normalizing gene for the stable expression in 786-O and 769-P cells under hypoxic condition [27]. *MAT2A*, *AHCY*, and *BHMT*, which are in charge of methionine conversion, were all dramatically diminished under hypoxia (Figure 1D and Figure S2A), suggesting that the enhanced methionine metabolism is not due to increased enzyme expression. Subsequently, we further examined the expression of transporters charged for methionine or folate in 786-O and 769-P cells cultured under normoxia and hypoxia, respectively. The mRNA expression of folate transporters (*PCFR*, *RFC*, and *FR*) were diminished (Figure S1A), while methionine uptake transporters (*LAT1*, *LAT3*, and *LAT4*) were increased under hypoxia (Figure 1E and Figure S2B). Among them, L-type amino acid transporter 1 (LAT1) upregulated noticeably compared with others under hypoxia. In addition, the protein expression of LAT1 also markedly upregulated in 786-O and 769-P cells under hypoxia compared with normoxia (Figure 1F). Furthermore, compared with normoxic conditions, the mRNA expression of LAT1 in RCC cell lines (769-P and 786-O) was increased and presented a time-dependent manner in the anoxic conditions (Figure 1G), implying that LAT1 expression would be induced by hypoxia in RCC cells. Generally, LAT1 forms a heteromeric complex with 4F2 cell-surface antigen-heavy chain (4F2hc, encoded by *SLC3A2*) and the complex is responsible for transporting amino acids [28]. Therefore, we also detected the expression of *SLC3A2* in 786-O and 769-P cells cultured under normoxia and hypoxia, respectively. Consistent with LAT1 expression, the expression of *SLC3A2* was significantly increased under hypoxia (Figure S3). Taken together, increased methionine metabolism would be regulated by upregulation of LAT1 in RCC cells under hypoxic conditions.

### 3.2. Upregulated LAT1 Mediates Methionine Accumulation in RCC Cells under Hypoxia

**Figure 2:**
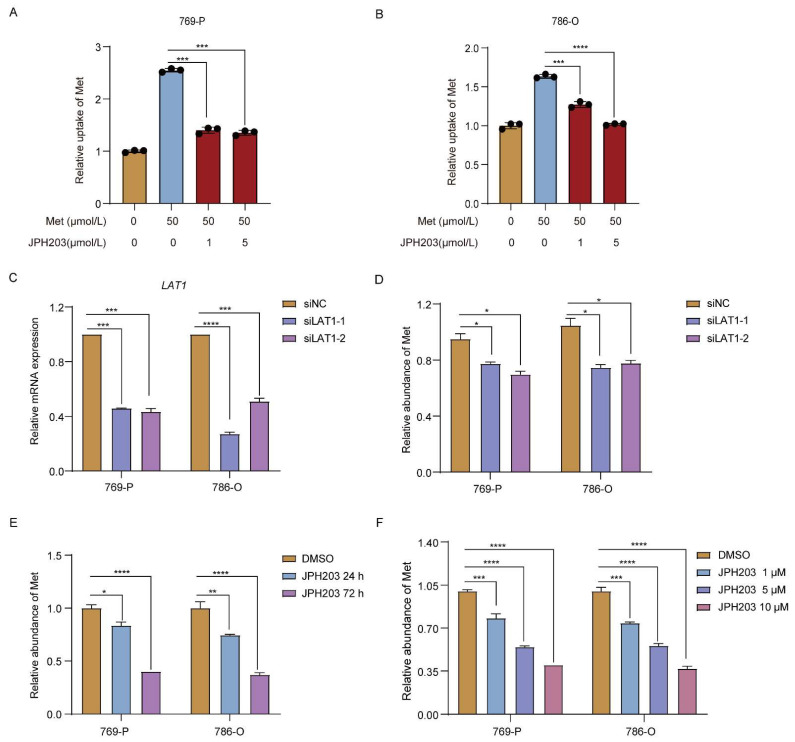
LAT1 regulates methionine accumulation in RCC cells. (**A**,**B**) The uptake of methionine was detected by LC-MS in 786-O (**A**) and 769-P (**B**) after being incubated with 50 µmol/L methionine with JPH203 (0, 1, and 5 µmol/L) at 37 °C for 3 min. (**C**) Expression of *LAT1* in 786-O and 769-P cells after being treated with LAT1 specific siRNAs. *GAPDH* was used as the normalizing gene. (**D**) The methionine accumulation in 786-O and 769-P cells after being treated with LAT1 specific siRNAs for 48 h. (**E**) Methionine accumulation in 786-O and 769-P cells after being treated with 10 µmol/L JPH203 for 24 h and 72 h, respectively. (**F**) Intracellular methionine accumulation in 786-O and 769-P cells after being treated with JPH203 (0, 1, 5, and 10 µmol/L) for 72 h. Met—Methionine. Data are the mean ± SEM for biological triplicates. One-way ANOVA analysis was used. * *p* < 0.05; ** *p* < 0.01; *** *p* < 0.001; **** *p* < 0.0001.

To further assess the contribution of LAT1 for methionine accumulation under hypoxia, LAT1 specific short-interfering RNA (siRNA) and LAT1 inhibitor JPH203 were used to knock down LAT1 expression and block LAT1 activity, respectively. Then, the abundance of intracellular methionine in RCC cells (786-O and 769-P) was detected using LC-MS. As displayed in Figure 2A,B, the uptake of methionine was significantly decreased in 786-O and 769-P cells after being incubated with 50 µmol/L methionine with JPH203 (1 and 5 µmol/L) at 37 °C for 3 min, suggesting that LAT1 take charge of uptake of methionine in RCC cells. Furthermore, the accumulation of methionine was markedly lessened in both 786-O and 769-P cells after knockdown of LAT1 expression by siRNAs or inhibition of LAT1 activity by JPH203, respectively (Figure 2C–F). Moreover, the diminishment of methionine accumulation by JPH203 presented a time- and concentration-dependent manners in RCC cell lines (Figure 2E,F). The accumulation of methionine in 786-O and 769-P cells decreased significantly with increasing time of JPH203 treatment (24, and 72 h) and concentrations of JPH203 (0, 1, 5, and 10 µmol/L). Taken together, the upregulation of LAT1 could increase methionine uptake, resulting in increased methionine accumulation in RCC cells under hypoxic conditions.

### 3.3. LAT1 Could Be an Indicator for Grades and Overall Survival of RCC

**Figure 3:**
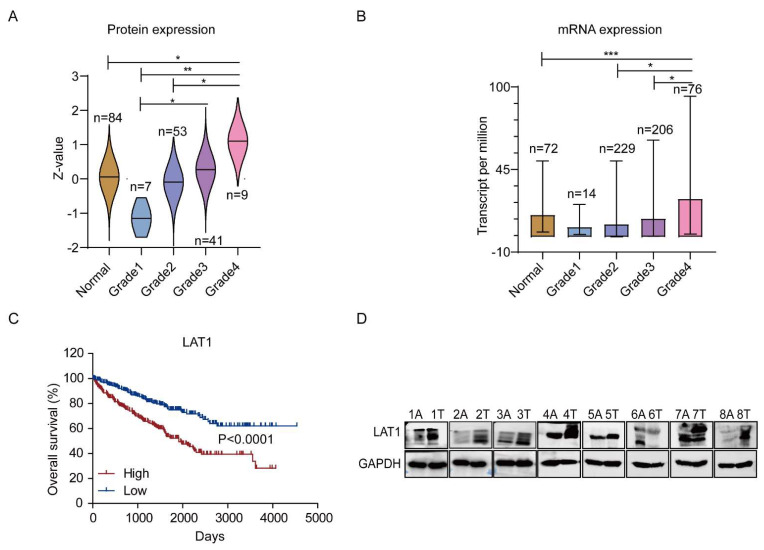
Clinical relevance of LAT1 expression to RCC. (**A**) The protein expression of LAT1 in RCC patients with different clinical stages. The data acquired from CPTAC database. Z values represent standard deviations from the median across samples for RCC. (**B**) The mRNA expression of LAT1 in RCC patients with different clinical stages. The data acquired from TCGA database. (**C**) Kaplan–Meier survival analysis of RCC patients with high and low LAT1 expression. (**D**) The protein expression of LAT1 in samples of matched RCC and adjacent non-tumor. A—adjacent tissue; T—tumor. GAPDH was used as an internal control. One-way ANOVA analysis was used. * *p* < 0.05; ** *p* < 0.01; *** *p* < 0.001.

Given the close relation between the hypoxic microenvironment and RCC progression [29], the overall survival and LAT1 expression for RCC patients was analyzed based on The Cancer Genome Atlas (TCGA) database [30]. As shown in Figure 3C, clinical patients of RCC (265 cases) with high LAT1 expression had a shorter overall survival. We further investigated correlation of LAT1 expression at mRNA and protein level and clinical grades of RCC from the database of TCGA and the Clinical Proteomic Tumor Analysis Consortium (CPTAC). LAT1 expression both in mRNA and protein strengthened with increasing of clinic grades of RCC (Figure 3A,B). Moreover, to verify the results from database analysis, we also performed LAT1 evaluation in clinical samples of RCC tumor. The protein expression of LAT1 in the tumor tissue was significantly higher than that in adjacent tissue (Figure 3D). These results not only implies that LAT1 is closely related to the worsening of RCC, but also suggests that LAT1 could be a marker for the development of RCC.

### 3.4. LAT1 Mediates Oxaliplatin Accumulation in RCC Cells by Inhibiting GSH Production

**Figure 4:**
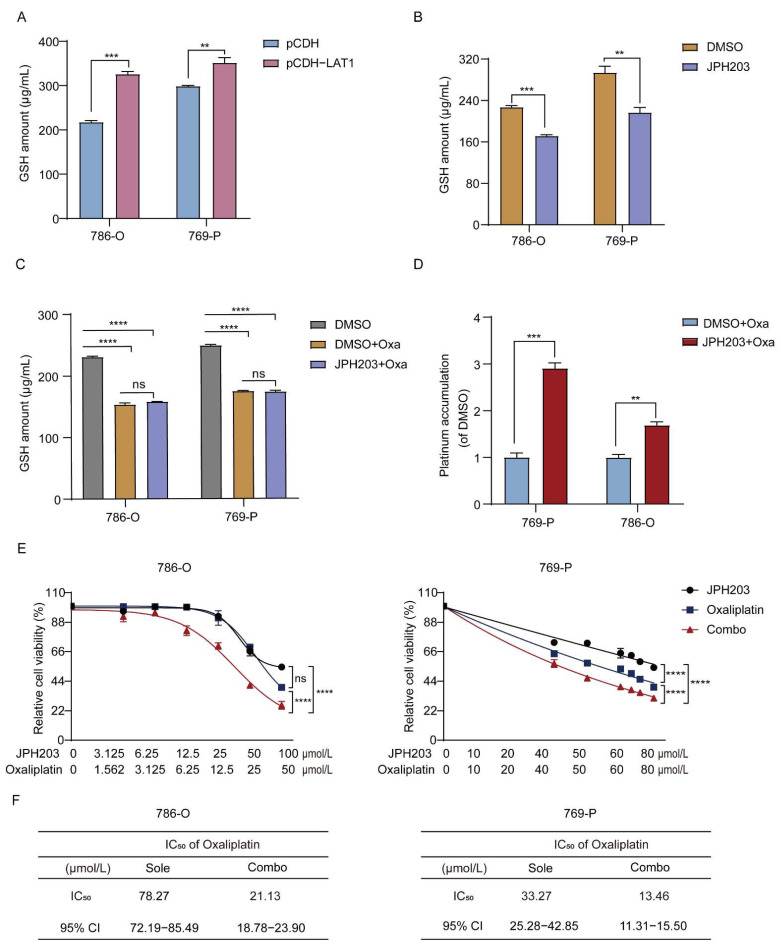
LAT1 inhibitor increases oxaliplatin accumulation via inhibiting GSH synthesis. (**A**) The amount of intracellular GSH in RCC cells (786-O and 769-P) with LAT1 overexpression (versus vector). (**B**) The amount of intracellular GSH in RCC cells (786-O and 769-P) after being treated with 10 µmol/L JPH203 (versus DMSO). (**C**) The accumulation of GSH in RCC cells (786-O and 769-P) treated with oxaliplatin (0 and 50 µmol/L) with and without JPH203 (10 µmol/L). (**D**) Oxaliplatin accumulation was detected by ICP−MS in 786-O and 769-P cells treated with JPH203 (0 and 10 µmol/L) for 48 h followed by adding 50 µmol/L oxaliplatin for 48 h. (**E**) The cell viability of 786-O and 769-P cells treated with JPH203 with and without oxaliplatin. Combo—JPH203 in combination with oxaliplatin. (**F**) IC_50_ values of oxaliplatin in RCC cells (769-P and 786-O) undergoing oxaliplatin alone or combination treatment. Oxa—oxaliplatin. Data are the mean ± SEM for biological triplicates. Students *t*-test (two-tailed), one-way ANOVA analysis, and two-way ANOVA analysis were used. ** *p* < 0.01; *** *p* < 0.001; **** *p* < 0.0001. ns—no significance.

Methionine could convert to cysteine via metabolic pathway of methionine cycle and transsulfuration, which therefore is in close correlation to glutathione (GSH) formation [17]. From this perspective, GSH synthesis would be induced by accumulated methionine in RCC cells. Moreover, coordination with GSH and then efflux out of cells to reduce intracellular drug concentration is an important pathway for tumors to be resistant to oxaliplatin [31]. Therefore, the increase in methionine accumulation, mediated by LAT1 upregulation, would promote oxaliplatin resistance via increasing GSH-mediated oxaliplatin consumption. To verify this hypothesis, the impact of LAT1 on GSH synthesis was elucidated firstly. After constructing the 786-O and 769-P cell lines with high LAT1 expression (Figure S4A), a positive correlation between GSH level and LAT1 expression was found in RCC cell lines (786-O and 769-P) with overexpression of LAT1 (Figure 4A). In contrast, the amount of GSH and methionine was reduced in RCC cells treated with JPH203 (Figure 4B and Figure S4B), implying that GSH production would be regulated by LAT1 via mediating methionine accumulation. Subsequently, the effects of LAT1 on GSH-mediated oxaliplatin excretion was examined. The accumulation of GSH was assessed in 786-O and 769-P cells after being treated with oxaliplatin and JPH203. As shown in Figure 4C, oxaliplatin significantly reduced the GSH content in cells, indicating that GSH consumption caused by chemical chelation occurred in cells. In addition, GSH content in DMSO + Oxa and JPH203 + Oxa groups did not show a significant difference, manifesting that JPH203 caused less GSH consumption, which would result more oxaliplatin accumulated in cells. Therefore, the intracellular oxaliplatin accumulation was further estimated. As displayed in Figure 4D, the accumulation of oxaliplatin in 786-O and 769-P was significantly increased after JPH203 treatment, indicating that combination of JPH203 and oxaliplatin have the potential to enhance efficacy against RCC cells.

Transporter plays an important role in oxaliplatin uptake and intracellular accumulation [32]. Thus, we wanted to clarify whether JPH203 is involved in the regulation of oxaliplatin transporters. Organic cation transporter 2 (OCT2) has been regard as a most arrestive member of oxaliplatin uptake transporters in renal tubule epithelial cells [33]. The activity of OCT2 can generally be presented through the uptake of model substrate 1-methyl-4-phenylpyridinium (MPP^+^). As displayed in Figure S5A, JPH203 significantly decreased the uptake of MPP^+^, implying that JPH203 would inhibit the activity of OCT2. Therefore, the accumulation of oxaliplatin in RCC cells after JPH203 treatment is unlikely to be the result of OCT2 activation. In addition, other uptake transporters (OCTN1, OCTN2, CTR1, and OCT3) and efflux transporters (MATE-2K), which take charge for the transportation of oxaliplatin to some extent, were also evaluated via mRNA expression [32]. The result show that there is no significant difference between with and without JPH203 treatment (Figure S5B). Collectively, increased intracellular accumulation of oxaliplatin by JPH203 is not achieved through the pathway of the oxaliplatin transport regulation.

We further examined the effects of combination strategy on oxaliplatin sensitivity of RCC cells. The viability of 786-O and 769-P cells was evaluated using CCK-8 assay after treating with JPH203, oxaliplatin, and combination strategy, respectively. As displayed in Figure 4E, the cell viability both in 786-O and 769-P cells was inhibited more significantly under the combined treatment when compared with solely JPH203 or oxaliplatin. JPH203 and oxaliplatin combination presented a synergetic effect by calculation of combination index (<1) (Figure S6). In addition, the IC_50_ of combination with JPH203 was significantly lowered both in 786-O and 769-P (78.27 vs. 21.13 µmol/L and 33.27 vs. 13.46 µmol/L) compared with sole oxaliplatin (Figure 4F). Collectively, these results indicated that LAT1 inhibition sensitized RCC cells to oxaliplatin, which would be an effective way to address resistance to platinum-based drugs for RCC.

### 3.5. LAT1 Inhibitor Combination with Oxaliplatin Potentiates Antitumor Efficiency In Vivo

**Figure 5:**
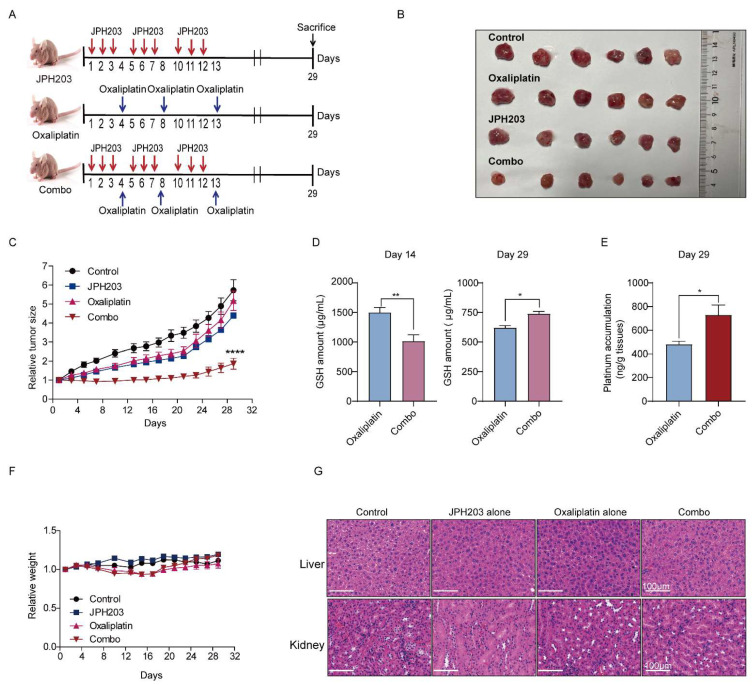
The combination of oxaliplatin and JPH203 enhance the therapeutic effect of RCC tumors. (**A**) The timeline of drug administration. (**B**) Representative photo of the tumors was stripped from sacrificed mice. (**C**) Relative tumor growth of mice bearing drug administration (either single drug or in combination). (**D**) The amount of GSH was detected in tumor of mice bearing with oxaliplatin with and without JPH203 at day 14 and day 29, respectively. (**E**) ICP-MS was used to detect platinum accumulation in tumor tissues from mice treated with oxaliplatin with and without JPH203 at day 29. (**F**) The curves of weight in mice bearing different treatment. (**G**) The hematoxylin–eosin staining was used to evaluate for toxicity of livers and kidneys in mice bearing different drug treatments. Scale bar = 100 µm. Combo—JPH203 in combination with oxaliplatin. Data represent the mean ± SEM. Students t-test (two-tailed) and one-way ANOVA analysis were used. * *p* < 0.05; ** *p* < 0.01; **** *p* < 0.0001.

After demonstrating the efficacy of the combination of LAT1 inhibitor JPH203 and oxaliplatin at the cellular level, we further evaluated the effect of the combination strategy in vivo using 786-O xenograft mouse models. As the animal experiment protocol presented in Figure 5A, the model mice were sacrificed, and we stripped the tumors at day 29. The tumor size in the combination group were relatively smaller compared with JPH203 and oxaliplatin alone (Figure 5B). Further statistics on the size of RCC tumor showed that the combination strategy presented a stronger therapeutic effect in terms of growth rate and final tumor volume throughout the treatment duration (Figure 5C). Moreover, intertumoral GSH and platinum accumulation was further analyzed. In the combination group, GSH consumption was lowered while platinum was elevated compared with oxaliplatin group (Figure 5D,E). In addition, we found that the combination strategy displayed a good biocompatibility, evidencing by that the weight and vital organs (liver and kidney) of the mouse model present no significant difference to the control group by evaluation of weight, biochemical indicators in blood, and tissue section (hematoxylin–eosin staining) (Figure 5F,G and Figure S7). Taken together, the combination of oxaliplatin and LAT1 inhibitor (JPH203) significantly enhanced the therapeutic effect of RCC tumors, which provides a potential solution for chemotherapy resistance for solid tumor.

## 4. Discussion

Methionine as the essential amino acids participated in one-carbon-unit metabolism, folate cycle, polyamine, glutathione, cysteine, and nucleotide synthesis, which would be involved the occurrence, development, metastasis, and chemoresistance of tumors by interfering with gene expression or epigenetic modification [34,35]. Numerous studies have confirmed that the proliferation rate of tumor cells is dependent on the uptake of exogenous methionine, which is also known as the “Hoffman effect” [22]. Aiming for this mechanism, methionine-restriction therapy, represented by reducing the uptake of exogenous methionine [36] and oral recombinant aminotransferase [37], has been proposed. However, systemic restriction of methionine would lead to overall abnormalities in the body, including dysfunction of gut flora and T cells [38]. Hence, specific restriction of methionine metabolism in the tumor cells is important for the development of RCC therapy. 

LAT1, a sodium-independent amino acid transporter, would form a heterodimer with 4F2 cell-surface antigen-heavy chain (4F2hc), which is responsible for the uptake of neutral amino acids. Intensive studies demonstrated that the LAT1 overexpression not only promotes cell proliferation and migration via activating mTORC1 pathway in various cancers [39,40], but also mediated methionine cycle in lung tumor cells [41]. Our work revealed that upregulated LAT1 under hypoxia promotes methionine accumulation and metabolism, leading to GSH accumulation in RCC cells. Therefore, we confirmed that LAT1 and the uptake and metabolism of methionine are closely related in RCC under hypoxia, but the question of whether LAT1 affects the development of RCC through other mechanisms (e.g., meditation of immune cell activation [42,43]) needs to be further elucidated. 

Oxaliplatin aberrates DNA replication and transcription, presenting an excellent therapeutic effect in tumors, including colorectal cancer, ovarian cancer, and RCC [44,45,46]. However, the therapeutic efficacy would be significantly weakened in hypoxic environment, causing chemotherapy resistance to gradually occur [47]. In this work, we found that the combined LAT1 inhibitor JPH203 enhanced the therapeutic efficacy of RCC by increasing the amount of intracellular oxaliplatin. This effect may be achieved by restricting GSH synthesis after blocking LAT1. According to previous studies, platinum drugs, including oxaliplatin, could accelerate extracellular excretion through chelation with GSH to reduce the effective drug concentration in cells, thus showing reduced therapeutic efficacy on tumor cells [48,49]. Therefore, combination of JPH203 and oxaliplatin exhibits a synergistic effect on RCC treatment both in vitro and in vivo because JPH203 restricts GSH generation through obstructing methionine metabolism and impairing chelation with oxaliplatin. Overall, our work explained the potential mechanism of methionine-limiting therapy, as well as provided a theoretical and experimental basis for exploitation of novel drug combination strategies.

## 5. Conclusions

Overall, we proposed a combination strategy of an LAT1 inhibitor and oxaliplatin that remains effective and stable under a hypoxic environment for RCC treatment. We also elucidated that LAT1 inhibitors limit the binding of oxaliplatin to GSH through obstructing methionine metabolism, thereby increasing intracellular drug content, resulting in decreased drug tolerance and synergistic effects. This work promotes the development of RCC treatments, and provides new insights into the combined strategy exploitation for solid tumors with hypoxic microenvironments.

## 6. Patents

The patent application number: 202210219957.X.

## Data Availability

The data presented in this study are available on request from the corresponding author.

## Supplementary Materials

The following supporting information can be downloaded at: https://www.mdpi.com/article/10.3390/cancers14102551/s1, Figure S1: The effect of hypoxia on folate metabolism. (**A**) The mRNA expression of folate transporters (*RFC*, *FR*, and *PCFT*) in 769-P cells exposed in hypoxic conditions and normoxic conditions for 72 h, respectively. RFC—reduced folate carrier; PCFT—proton-coupled folate transporter; FR—folate receptor. (**B**) The mRNA expression of enzymes (*SHMT1*, *SHMT2*, and *MTFHR*) of folate cycle in 786-O and 769-P cells cultured under normoxia and hypoxia for 72 h, respectively. N—normoxia; H—hypoxia. *GAPDH* was used as the normalizing gene. Data are the mean ± SEM for biological triplicates. Students t-test (two-tailed) was used. * *p* < 0.05; ** *p* < 0.01, Figure S2: Expression level of enzymes and transporters of methionine cycle in RCC cells under hypoxia. (**A**) The mRNA expression of pivotal enzymes (*MAT2A*, *AHCY*, *BHMT*, and *GNMT*) of methionine cycle in 786-O cells cultured under normoxia and hypoxia for 72 h, respectively. MAT2A—methionine adenosyltransferase 2A; GNMT—glycine N-methyltransferase; AHCY—adenosylhomocysteinase; BHMT—betaine-homocysteine S-methyltransferase. (**B**) The mRNA expression of methionine uptake transporters (*LAT1*, *LAT2*, *LAT3*, and *LAT4*) in 786-O cells cultured under normoxia and hypoxia for 72 h, respectively. LAT—L-type amino acid transporter. N—normoxia; H—hypoxia. *GAPDH* was used as the normalizing gene. Data are the mean ± SEM for biological triplicates. Students t-test (two-tailed) was used. * *p* < 0.05; ** *p* < 0.01; **** *p* < 0.0001. ns—no significance, Figure S3: The mRNA level of *SLC3A2* in 786-O and 769-P cells cultured under hypoxia and normoxia for 72 h, respectively. *GAPDH* was used as the normalizing gene. Data are the mean ± SEM for biological triplicates. Students t-test (two-tailed) was used. * *p* < 0.05; ** *p* < 0.01, Figure S4: Efficiency of overexpression and inhibition of LAT1 in RCC cells. (**A**) The mRNA expression of LAT1 in 769-P and 786-O cells with LAT1 overexpression (versus vector). (**B**) Relative methionine uptake was examined using LC-MS in 769-P and 786-O cells treated with 10 µmol/L JPH203 (versus DMSO) for 72 h. Met—methionine. *GAPDH* was used as the normalizing gene. Data are the mean ± SEM for biological triplicates. Students t-test (two-tailed) was used. * *p* < 0.05; ** *p* < 0.01; **** *p* < 0.0001, Figure S5: The expression and uptake capacity of transporter of oxaliplatin in RCC cells after being treated with JPH203 (**A**) The uptake of MPP^+^ (1-methyl-4-phenylpyridinium) was examined in 769-P cells after being treated with JPH203 (0, 10, 50 µmol/L). (**B**) The mRNA expression of organic cation/carnitine transporters (OCTN1 and OCTN2), organic cation transporters (OCT2 and OCT3), high-affinity copper uptake protein (CTR1), and multidrug and toxin extrusion protein (MATE-2K) in 769-P cells after being treated with 10 µmol/L JPH203. *GPADH* was used as the normalizing gene. Data are the mean ± SEM for biological triplicates. Students t-test (two-tailed) was used. * *p* < 0.05; ** *p* < 0.01. ns—no significance, Figure S6: Combination index–fraction-affected plots of JPH203 and oxaliplatin combinations in 786-O and 769-P cells. Combination index (CI) < 1 represents synergism, Figure S7: The biocompatibility of combination strategy. (**A**) The amount of AST (aspartate aminotransferase), ALT (alanine transaminase), and ALB (albumin) in plasma of mice bearing different drug treatment. (**B**) The amount of CREA (creatinine) and BUN (blood urea nitrogen) in plasma of mice bearing different drug treatment. Combo—JPH203 in combination with oxaliplatin. Data are the mean ± SEM for biological triplicates. One-way ANOVA analysis was used. * *p* < 0.05; ** *p* < 0.01; ns, no significance, Figure S8: Original Western blots of Figure 1F and Figure 3D, Table S1: Primers used in this study, Table S2: Tissue specimen information.
