# Supplementary material for: Blockade LAT1 Mediates Methionine Metabolism to Overcome Oxaliplatin Resistance under Hypoxia in Renal Cell Carcinoma
Source: Cancers (Basel). 2022 May 22;14(10):2551. doi: 10.3390/cancers14102551 (PMC9139506; doi:10.3390/cancers14102551)
Supplement: Supplementary file 1 [file cancers-14-02551-s001.zip › cancers-1708720-supplementary.pdf]

Supplementary Materials: Blockade LAT1 Mediates Methionine Metabolism to Overcome Oxaliplatin Resistance under Hypoxia in Renal Cell Carcinoma

Qingwen Xu, Yuxi Liu, Wen Sun, Tiantian Song, Xintong Jiang, Kui Zeng, Su Zeng, Lu Chen and Lushan Yu

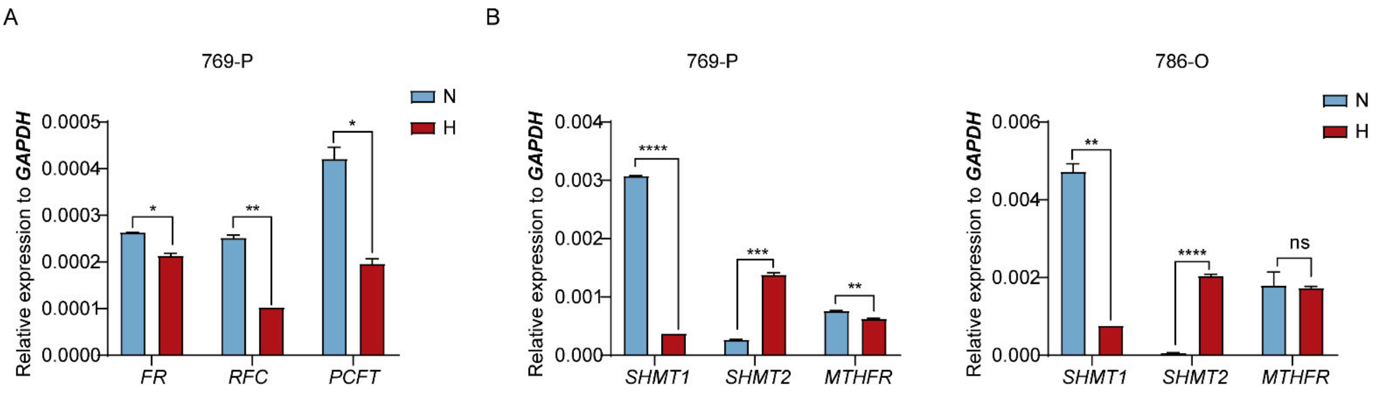

Figure S1. The effect of hypoxia on folate metabolism. (**A**) The mRNA expression of folate transporters (*RFC*, *FR*, and *PCFT*) in 769-P cells exposed in hypoxic and normoxic conditions for 72 h, respectively. RFC: Reduced folate carrier; PCFT: Proton-coupled folate transporter; FR: Folate receptor. (**B**) The mRNA expression of enzymes (*SHMT1*, *SHMT2*, and *MTHFR*) of folate cycle in 786-O and 769-P cells cultured under normoxia and hypoxia for 72 h, respectively. N, normoxia; H, hypoxia. *GAPDH* was used as the normalizing gene. Data are the mean ± SEM for biological triplicates. Student's *t* test (two-tailed) was used. * $p < 0.05$; ** $p < 0.01$; *** $p < 0.001$; **** $p < 0.0001$. ns: no significance.

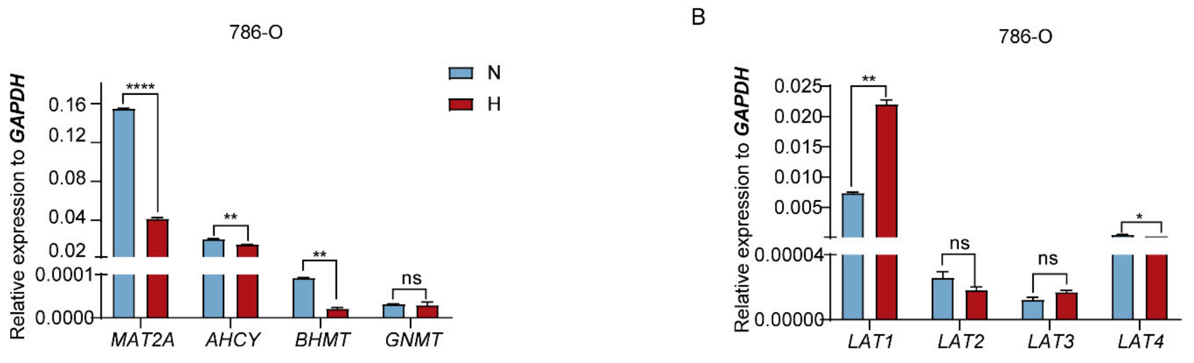

Figure S2. Expression level of enzymes and transporters of methionine cycle in RCC cells under hypoxia. (**A**) The mRNA expression of pivotal enzymes (*MAT2A*, *AHCY*, *BHMT*, and *GNMT*) of methionine cycle in 786-O cells cultured under normoxia and hypoxia for 72 h, respectively. MAT2A: Methionine adenosyltransferase 2A; GNMT: Glycine N-methyltransferase; AHCY: Adenosylhomocysteinase; BHMT: Betaine-homocysteine S-methyltransferase. (**B**) The mRNA expression of methionine uptake transporters (*LAT1*, *LAT2*, *LAT3*, and *LAT4*) in 786-O cells cultured under normoxia and hypoxia for 72 h, respectively. LAT: L-type amino acid transporter. N, normoxia; H, hypoxia. *GAPDH* was used as the normalizing gene. Data are the mean ± SEM for biological triplicates. Student's *t* test (two-tailed) was used. * $p < 0.05$; ** $p < 0.01$; **** $p < 0.0001$. ns: no significance.

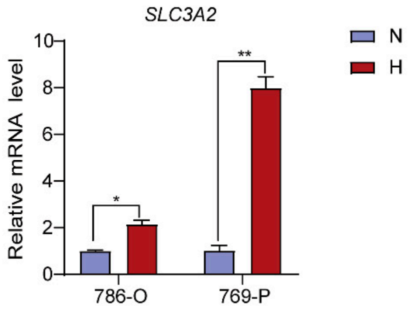

Figure S3. The mRNA level of *SLC3A2* in 786-O and 769-P cells cultured under normoxia and hypoxia for 72 h, respectively. N, normoxia; H, hypoxia. *GAPDH* was used as the normalizing gene. Data are the mean ± SEM for biological triplicates. Student's *t* test (two-tailed) was used. * $p < 0.05$; ** $p < 0.01$.

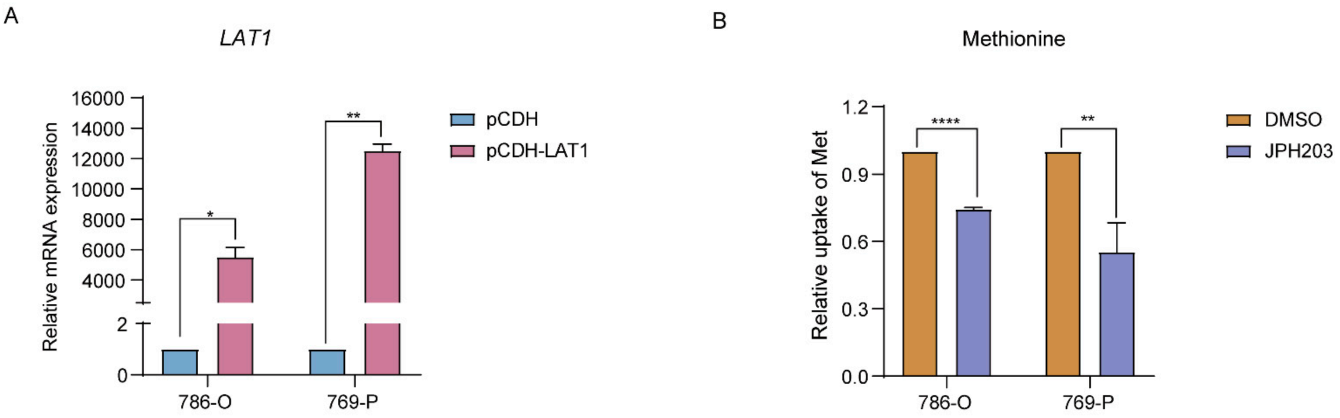

Figure S4. Efficiency of overexpression and inhibition of LAT1 in RCC cells. (**A**) The mRNA expression of *LAT1* in 769-P and 786-O cells with LAT1 overexpression (versus vector). *GAPDH* was used as the normalizing gene. (**B**) Relative methionine uptake was examined using LC-MS in 769-P and 786-O cells treated with 10 µmol/L JPH203 (versus DMSO) for 72 h. Met: methionine. Data are the mean ± SEM for biological triplicates. Student's *t* test (two-tailed) was used. * $p < 0.05$; ** $p < 0.01$; **** $p < 0.0001$.

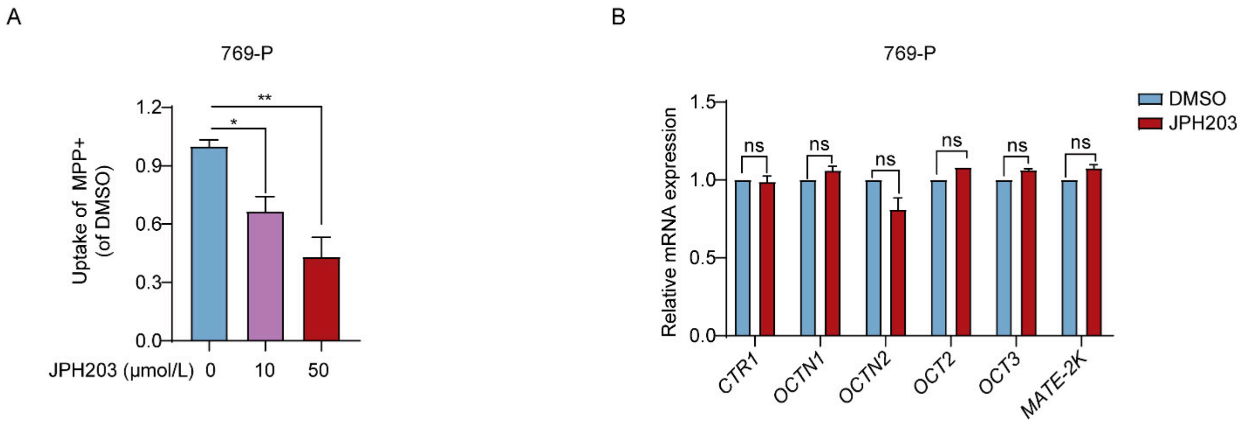

Figure S5. The expression and uptake capacity of transporter of oxaliplatin in RCC cells after being treated with JPH203 (**A**) The uptake of MPP^+ (1-methyl-4-phenylpyridinium) was examined in 769-P cells after being treated with JPH203 (0, 10, 50 µmol/L). (**B**) The mRNA expression of organic cation/carnitine transporters (*OCTN1* and *OCTN2*), organic cation transporters (*OCT2* and *OCT3*), high affinity copper uptake protein (*CTR1*), and multidrug and toxin extrusion protein (*MATE-2K*) in 769-P cells after being treated with 10 µmol/L JPH203. *GPADH* was used as the normalizing gene.

Data are the mean ± SEM for biological triplicates. Student's *t* test (two-tailed) was used. * $p < 0.05$; ** $p < 0.01$. ns: no significance.

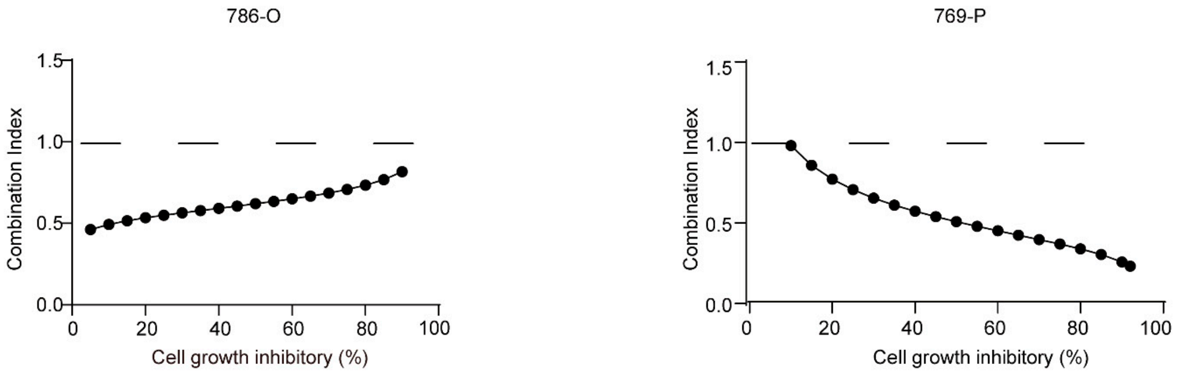

Figure S6. Combination index–fraction affected plots of JPH203 and oxaliplatin combinations in 786-O and 769-P cells. Combination index (CI) < 1 represents synergism.

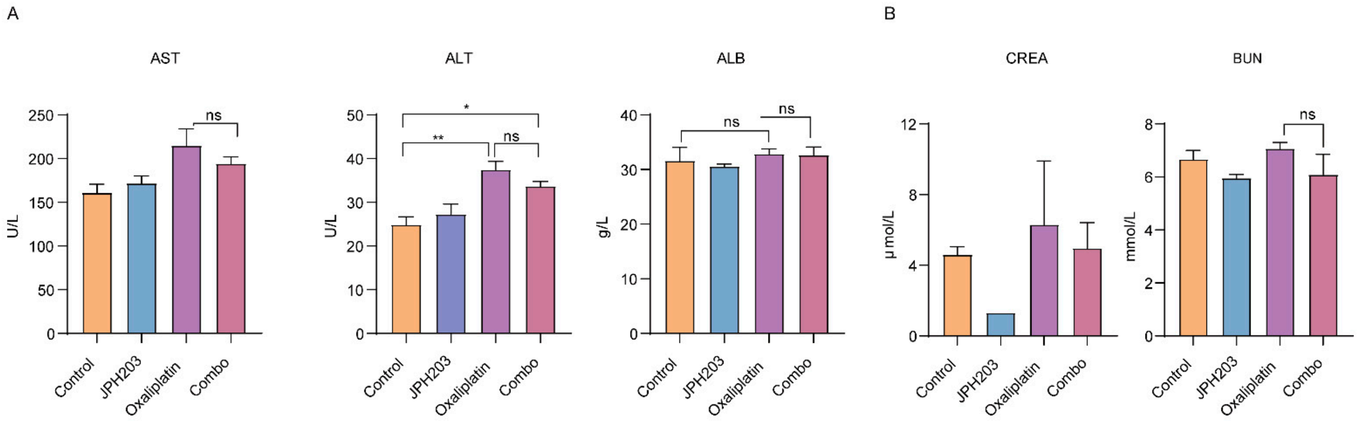

Figure S7. The biocompatibility of combination strategy. (**A**) The amount of AST (Aspartate aminotransferase), ALT (Alanine transaminase), and ALB (Albumin) in plasma of mice bearing different drug treatment. (**B**) The amount of CREA (Creatinine) and BUN (Blood urea nitrogen) in plasma of mice bearing different drug treatment. Combo: JPH203 in combination with oxaliplatin. Data are the mean ± SEM for biological triplicates. One-way ANOVA analysis was used. * $p < 0.05$; ** $p < 0.01$; ns, no significance.

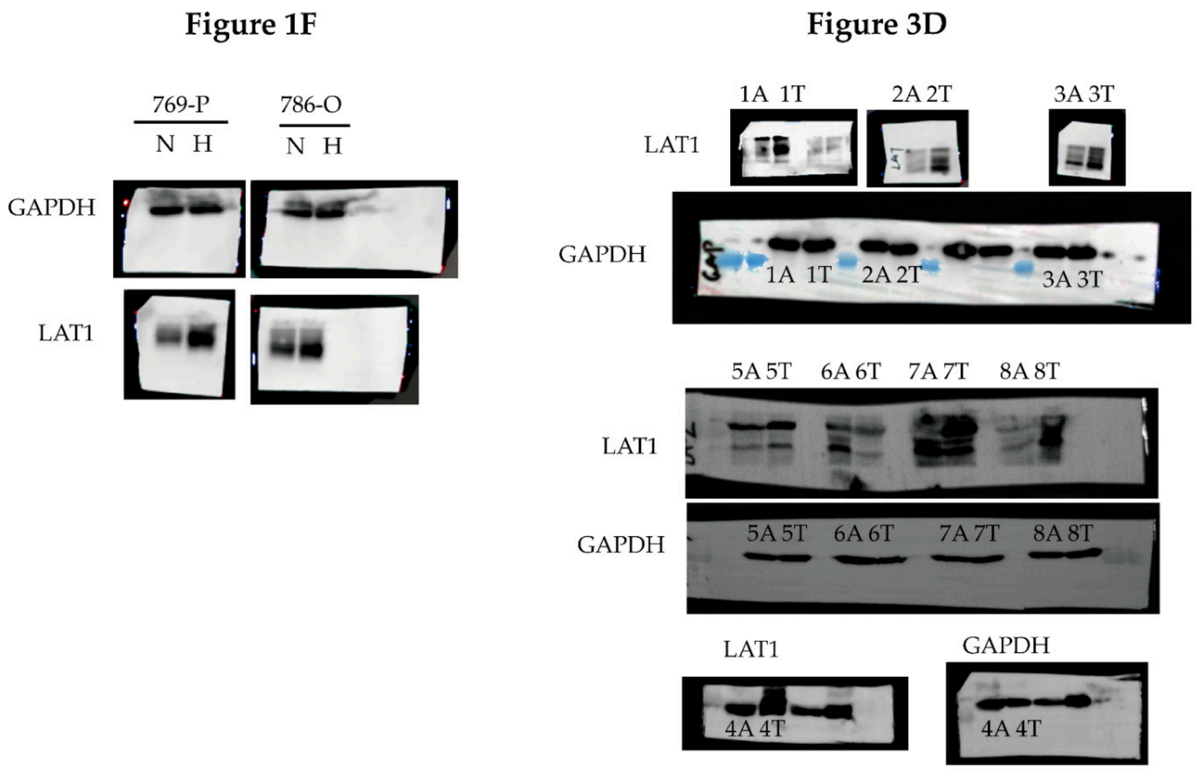

Figure S8. Original western blots of Figure 1F and Figure 3D.

Table S1. Primers used in this study.

Gene	Forward (5′ to 3′)	Reverse (5′ to 3′)
GAPDH	AGGTGAAGGTCGGAGTCA	GGTCATTGATGGCAACAA
LAT1	GCATCGGCTTCACCATCATC	ACCACCTGCATGAGCTTCTGAC
PCFT	AGAGCTGGACAATGGATCGGT	GCCTTGCTGATAGCCATGACTC
RFC	GGGCTTTGTTGCTGGAAG	GGCAGAAAGGATTTGTCTCAAG
SHMT1	CGCAGAGTGCACCTTCCTGA	CACTGGTTCGAAGCTGCCTA
SHMT2	CTGCAGGAAGACCCTCTTGT	CGGAGTAGGGCTGGACATTG
AHCY	TAGCAGGCTATGGTGATGTGG	ATGGGGTCAATCTCGGTGATG
MTHFR	CCGCCGTGAACTACTGTGG	AGATGGCCCGTGATCTCCTC
MAT2A	ACCAGAAAGTGGTTCGTGAAG	CAAGGCTACCAGCACGTTACA
GNMT	GTATATCGGAGAGACACCCGCAG	CACTCTGGTCCCCTTTGCAG
BHMT	TGCTGGAGAGATTGTGATTGGA	CTTGTCTTCACTCGCATAGAAGG
FR	TTAGCCTGGCCCTAATGCT	GCAGGGATTTCCAGGTATCA

Table S2. Tissue specimen information.

Number	Gender	Age	Subtype	TNM stage
1	Male	37	Clear cell renal cell carcinoma	T1bN0M1
2	Male	70	Papillary cell renal cell carcinoma	T1bN0M0
3	Male	71	Clear cell renal cell carcinoma	T4aN2M0
4	Female	51	Clear cell renal cell carcinoma	T2aN0M0
5	Female	43	Clear cell renal cell carcinoma	T1aN0M0
6	Male	58	Clear cell renal cell carcinoma	T1bN0M0
7	Male	50	Clear cell renal cell carcinoma	T3N0M0
8	Male	52	Clear cell renal cell carcinoma	T4N1M0
